# Clinical Correlates of Type 2 Diabetes Self-Management in Adults: A Cross-Sectional Study

**DOI:** 10.3390/diseases14090348

**Published:** 2026-09-19

**Authors:** Paula-Alexandra Popovici, Andreea Diana Igna, Bianca-Lăcrimioara Petca, Timea Claudia Ghitea, Mihaela Simona Popoviciu

**Affiliations:** 1 Doctoral School of Biological and Biomedical Sciences, University of Oradea, 410087 Oradea, Romania; cipleu.paulaalexandra@student.uoradea.ro (P.-A.P.); igna.andreeadiana@student.uoradea.ro (A.D.I.); criste.biancalacrimioara@student.uoradea.ro (B.-L.P.); 2 Pharmacy Department, Faculty of Medicine and Pharmacy, University of Oradea, 410087 Oradea, Romania; 3 Department of Preclinical Disciplines, Faculty of Medicine and Pharmacy, University of Oradea, 410087 Oradea, Romania; mihaela.popoviciu@didactic.uoradea.ro

**Keywords:** type 2 diabetes, diabetes self-management, DSMQ, hyperglycemia, patient education, cross-sectional study, robust regression

## Abstract

Background: Diabetes self-management is essential for type 2 diabetes care, but cross-sectional associations may be distorted when questionnaire scores are incorrectly derived or converted into arbitrary categories. We evaluated clinical and sociodemographic correlates of the continuous Diabetes Self-Management Questionnaire (DSMQ) total score in a secondary analysis of an outpatient clinical database. Methods: The analysis included 262 adults with type 2 diabetes recruited between 1 November 2025 and 1 May 2026. DSMQ total and subscale scores were independently recalculated from the 16 item-level responses using the published scoring algorithm. The primary analysis retained the DSMQ score as a continuous outcome. The database provided a participant-level count of documented glucose measurements > 250 mg/dL. This operational count was not treated as a standardized clinical event or severity category; individual measurement timestamps, participant-specific observation duration, and the total number of glucose measurements were unavailable. Results: The recalculated DSMQ score was 5.26 ± 2.35 (median 5.10; interquartile range 3.12–7.08). Among 261 participants with an interpretable sex code, 144 (55.2%) were women. In univariable analyses, lower DSMQ scores were concurrently associated with longer diabetes duration, a higher recorded severe-hyperglycemia count, higher HbA1c, and higher fasting glucose. In the adjusted model (*n* = 261), the recorded severe-hyperglycemia count remained negatively associated with DSMQ score (adjusted coefficient −0.412 points per recorded episode; 95% confidence interval −0.621 to −0.203; *p* < 0.001). Individualized versus hospital-based education was not independently associated with the corrected score. A beta-regression sensitivity analysis produced a concordant direction of association (coefficient −0.152 on the logit mean scale; *p* < 0.001). The linear model explained 17.2% of observed variance (adjusted R^2^ = 0.136), with an optimism-corrected R^2^ of 0.099. Conclusions: The recorded severe-hyperglycemia count was a concurrent correlate of lower DSMQ score. Because the study is cross-sectional and glucose-measurement counts were preaggregated without observation-time or monitoring-frequency data, the findings do not establish temporal direction, causality, or individual-level clinical prediction. Item-level score verification and continuous-outcome analysis materially changed the interpretation of the dataset.

## 1. Introduction

Effective management of type 2 diabetes mellitus (T2DM) requires sustained patient engagement with medication use, nutrition, physical activity, glucose monitoring, and health-care contact. Inadequate engagement is associated with poorer metabolic outcomes and greater health-care use, although the determinants of self-management are heterogeneous and context-dependent [1,2].

The Diabetes Self-Management Questionnaire (DSMQ) was developed to assess self-care behaviors associated with glycemic control. Its 16 items generate four subscales—Glucose Management, Dietary Control, Physical Activity, and Health-Care Use—and a global Sum Scale transformed to a 0–10 metric, with higher scores representing more effective self-management [3]. The DSMQ therefore assesses a broader behavioral construct than medication adherence alone; using the two terms interchangeably can lead to construct misclassification [3,4].

Previous studies have linked DSMQ scores to glycemic control and have examined sociodemographic, educational, and clinical correlates of diabetes self-care [5,6,7]. Nevertheless, associations observed in cross-sectional data cannot establish temporal direction. Clinical burden may impair self-management, but poor self-management may also contribute to recurrent hyperglycemia. Treatment intensification and insulin use may similarly be markers of disease duration or severity rather than direct causes of poor self-management [8,9].

Analytical choices also influence inference. The DSMQ has no universally validated threshold for adequate self-management. Dividing a continuous score at the sample median discards information, creates sample-specific groups, and can destabilize effect estimates [10,11,12,13]. Accordingly, the present reanalysis retained the recalculated DSMQ total score as a continuous outcome. These findings should also be interpreted within the Romanian outpatient context, where national observational data have documented heterogeneous clinical and therapeutic profiles among adults with type 2 diabetes [12].

The primary objective was to identify clinical and sociodemographic correlates of the continuous DSMQ total score in adults with type 2 diabetes. The education variable was treated as a comparison of documented education modalities rather than as an intervention-effect estimate, because every participant had at least one recorded modality. Secondary objectives were to describe concurrent associations with glycemic measures, evaluate the internal consistency of recalculated DSMQ scales, assess model assumptions, and quantify explanatory performance and internal validity without implying temporal prediction.

## 2. Materials and Methods

### 2.1. Study Design, Setting, and Participants

This cross-sectional observational study was a secondary analysis of a CSV export from a prospectively maintained clinical database at a specialized diabetes and nutrition outpatient clinic in Romania. The source variables were collected during routine outpatient care and entered into the clinical database; the present work consisted of data cleaning, independent DSMQ rescoring, and statistical reanalysis of the export. Consecutive adults with established type 2 diabetes who completed the DSMQ were eligible. The documented recruitment window was 1 November 2025 to 1 May 2026. The supplied export did not contain the exact date of DSMQ administration, laboratory sampling, clinical consultation, or individual glucose measurements; therefore, the temporal order of these observations could not be established.

The CSV contained 263 rows. One row was an embedded header rather than a participant record and was excluded, leaving 262 participants with complete DSMQ item responses. One sex entry (“LM”) was uninterpretable and was treated as missing for sex-specific analyses and the multivariable model. One HbA1c value of 61% and one fasting-glucose value of 2293 mg/dL were treated as missing because the supplied export did not permit source-document verification. The primary regression used complete cases (*n* = 261); all other primary-model predictors had no missing values. Exploratory analyses involving HbA1c and fasting glucose used *n* = 261 for each variable. No prospective sample-size calculation was documented because this was a secondary analysis of the available dataset.

The term “reanalysis” refers to the correction of DSMQ scoring and refitting of the analyses during revision of the present manuscript using the same clinical database. It does not refer to a separate previously published study. The same data were analyzed using the stored derived DSMQ variables in an earlier version of this submission, but they have not been published or reported elsewhere.

### 2.2. Variables and Data Quality Procedures

Demographic, clinical, anthropometric, behavioral, laboratory, education, treatment, and DSMQ item variables were obtained from the database. Category labels were trimmed and harmonized only when spelling variants were unambiguous. Body mass index was recalculated as weight in kilograms divided by squared height in meters because the stored derived values were inconsistent with weight and height in several records. Missingness was limited to the uninterpretable sex code and the two implausible laboratory values described above. Because the CSV was not accompanied by source clinical documents, verification of those values and sensitivity analyses that retained them were not possible. Their exclusion did not affect the primary multivariable model because HbA1c and fasting glucose were not included as predictors.

The source database contained participant-level counts of documented glucose measurements < 54 mg/dL and >250 mg/dL. The >250 mg/dL cut-point was retained because it was the predefined threshold used to construct the exported database variable; it was not selected or modified during the present reanalysis. This operational cut-point should not be interpreted as a standardized definition of clinical severity or hyperglycemic crisis. Accordingly, the variable is described as the count of recorded glucose measurements > 250 mg/dL rather than as “recorded glucose measurements > 250 mg/dL. The export did not contain measurement-level timestamps, the number of glucose measurements per participant, or a rule for grouping consecutive measurements. Consequently, the recorded severe-hyperglycemia variable is a preaggregated count that could reflect both glycemic burden and monitoring intensity; repeated or closely spaced readings could not be deduplicated, and rates per observation time or proportions of measurements above the threshold could not be calculated. The hypoglycemia variable was handled analogously. Because the database did not record whether third-party assistance was required, level 3 hypoglycemia could not be separated from other low-glucose measurements. Because participant-specific observation duration and total measurement frequency were unavailable, the requested rate- and proportion-based sensitivity analyses could not be performed. The count may therefore reflect monitoring intensity as well as glycemic burden.

The two diabetes-education variables were almost mutually exclusive: 201 participants received hospital-based education only, 60 received individualized counseling only, and one received both; no participant had neither modality recorded. The source label “hospital-based education” was interpreted as structured diabetes education documented as delivered through the hospital program, not as education inferred solely from a hospital stay. The database did not specify curriculum, duration, or timing relative to DSMQ administration. For the regression, the single dual-modality participant was coded as 1 for the individualized-counseling indicator while retaining the hospital-education indicator; the reference group was hospital-based education only. Thus, the coefficient compares modalities and cannot estimate the presence, efficacy, or causal effect of education.

### 2.3. DSMQ Scoring and Outcome Definition

All DSMQ scores were independently recalculated from the item-level responses according to the published scoring algorithm [3]. The columns ending in “R” were treated as the raw responses to negatively worded items; these items (5, 7, 10, 11, 12, 13, 14, 15, and 16) were reverse-scored as 3 − response. The Glucose Management subscale used items 1, 4, 6, 10, and 12; Dietary Control used items 2, 5, 9, and 13; Physical Activity used items 8, 11, and 15; and Health-Care Use used items 3, 7, and 14. Item 16 contributed only to the total score. Each subscale sum was divided by its theoretical maximum and multiplied by 10; the total score was calculated as the 16-item sum divided by 48 and multiplied by 10. The pre-existing derived columns in the CSV were not used because they did not consistently reproduce the item-level calculation. Item numbers, domains, reverse-scoring decisions, and transformations are provided in Supplementary Table S1.

The primary outcome was the continuous recalculated DSMQ total score. A median-based classification was retained as a secondary methodological comparison rather than a conventional clinical sensitivity analysis; it is not a validated cut-off for adequate or inadequate self-management.

### 2.4. Statistical Analysis

Continuous variables are reported as mean ± standard deviation or median (interquartile range), as appropriate. Categorical variables are reported as number and percentage with the available denominator shown in Table 1. Internal consistency was assessed using Cronbach’s alpha after reverse scoring; subscale coefficients were interpreted in light of their small number of items. Univariable associations with the DSMQ total score were examined using Spearman correlations for continuous variables and Mann–Whitney tests with rank-biserial effect sizes for binary variables. Univariable *p*-values were considered exploratory; no formal multiplicity adjustment was applied, and interpretation emphasized effect sizes and confidence intervals.

The multivariable covariate set was defined during the present reanalysis, after correction of the DSMQ scoring procedure and before examination of the final regression results. Covariates were selected on clinical and methodological grounds and were not screened using univariable *p*-values. It included age, sex, years of education, urban residence, family support, diabetes duration, recalculated body mass index, hospitalizations in the previous year, the count of recorded glucose measurements > 250 mg/dL, education modality, and any insulin treatment. Self-monitoring of blood glucose and physical activity were not included because they overlap directly with DSMQ item content and could constitute part–whole adjustment. HbA1c and fasting glucose were treated as concurrent metabolic correlates rather than antecedent determinants. Binary-variable coding, including the dual-modality participant, is specified in Table 2 and Table 3.

Heteroskedasticity-consistent HC3 standard errors were used. Multicollinearity was assessed using variance inflation factors (VIFs). Linearity was assessed by inspection of residual-versus-fitted and partial-residual plots and by a Ramsey RESET test; influential observations were assessed with Cook’s distance. Because the DSMQ is bounded between 0 and 10, a beta-regression sensitivity analysis rescaled the score to (0, 1) using the Smithson–Verkuilen adjustment and refit the same predictor set. Model performance was summarized with R^2^, adjusted R^2^, and root-mean-square error (RMSE). Bootstrap internal validation used 1000 resamples of the complete-case dataset; in each resample the full model was refit, performance was calculated both in the resample and in the original complete-case data, and optimism was estimated as the mean difference. These procedures evaluate internal explanatory stability, not temporal prediction. Analyses were conducted in Python 3.11 using pandas 2.2.3, SciPy 1.15.2, statsmodels 0.14.4, and scikit-learn 1.5.2. Reporting was aligned with STROBE recommendations [14,15].

### 2.5. Ethical Considerations

This study was conducted in accordance with the Declaration of Helsinki and was approved by the Ethical Committee of the Faculty of Medicine and Pharmacy, University of Oradea (approval code 41; 31 October 2025). The present manuscript is a secondary analysis of the clinical and questionnaire data collected under that approved protocol. Informed consent covered research use of the clinical and DSMQ data. The analysis used a local working copy of the database, and published outputs contain aggregate, non-identifiable results only.

## 3. Results

### 3.1. Cohort Characteristics and Recalculated DSMQ Scores

The analysis included 262 participants. Mean age was 64.23 ± 10.97 years. Among 261 participants with an interpretable sex code, 144 (55.2%) were women. The median diabetes duration was 7.0 years (IQR 4.0–14.0), and recalculated mean body mass index was 31.70 ± 5.90 kg/m^2^. Hospital-based education only was recorded for 201 participants (76.7%); 61 participants (23.3%) received individualized counseling, including one who also had hospital-based education. Table 1 provides the available denominator for every variable.

**Figure 1:**
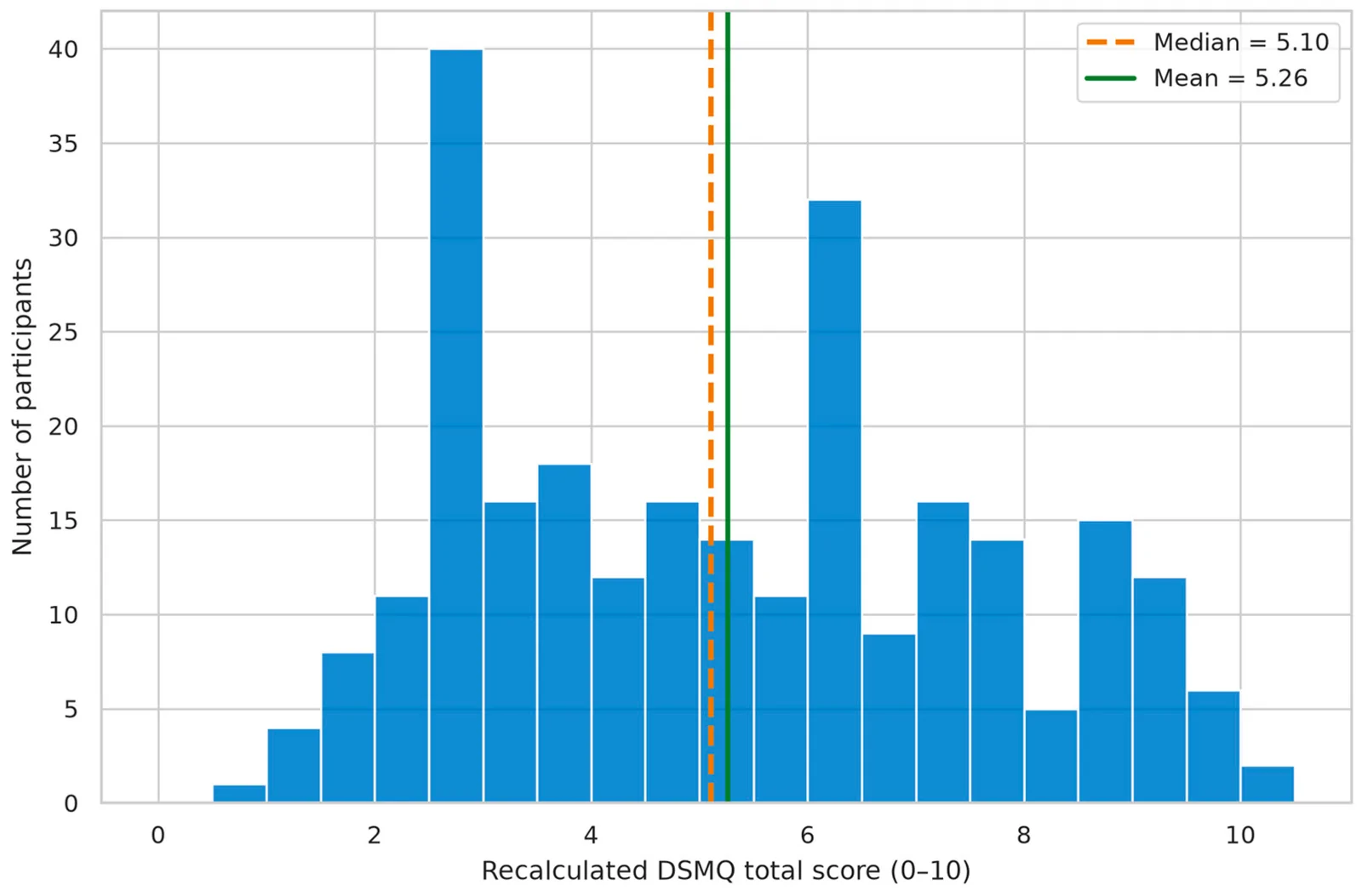
Distribution of the recalculated DSMQ total score. The orange dashed line shows the median (5.10), and the green solid line shows the mean (5.26). Because the score is calculated from 16 integer-coded items and transformed as (sum/48) × 10, only discrete score values in increments of approximately 0.21 are possible; the visible concentrations do not represent clinically defined participant subgroups.

The recalculated DSMQ total score was 5.26 ± 2.35, with a median of 5.10 (IQR 3.12–7.08) and a range of 0.63–10.00. No participant had a score at the lower boundary; two participants had a score of 10. Internal consistency was high for the total scale (Cronbach’s α = 0.916); subscale coefficients were 0.722 for Glucose Management, 0.892 for Dietary Control, 0.818 for Physical Activity, and 0.730 for Health-Care Use. These subscale coefficients should be interpreted in light of the three- or four-item subscale lengths (Figure 1).

### 3.2. Univariable Associations with the DSMQ Total Score

Higher DSMQ scores were weakly associated with more years of education (Spearman ρ = 0.150; *p* = 0.015) and urban residence (rank-biserial coefficient −0.211 under the stated coding; *p* = 0.003). Lower scores were associated with longer diabetes duration (ρ = −0.173; *p* = 0.005), a higher recorded severe-hyperglycemia count (ρ = −0.322; *p* < 0.001), higher HbA1c (ρ = −0.484; *p* < 0.001), higher fasting glucose (ρ = −0.371; *p* < 0.001), and insulin treatment (rank-biserial coefficient 0.236 under the stated coding; *p* = 0.001). These are unadjusted, concurrent associations; the signs of rank-biserial coefficients depend on the coding order, and none indicates causal direction.

**Figure 2:**
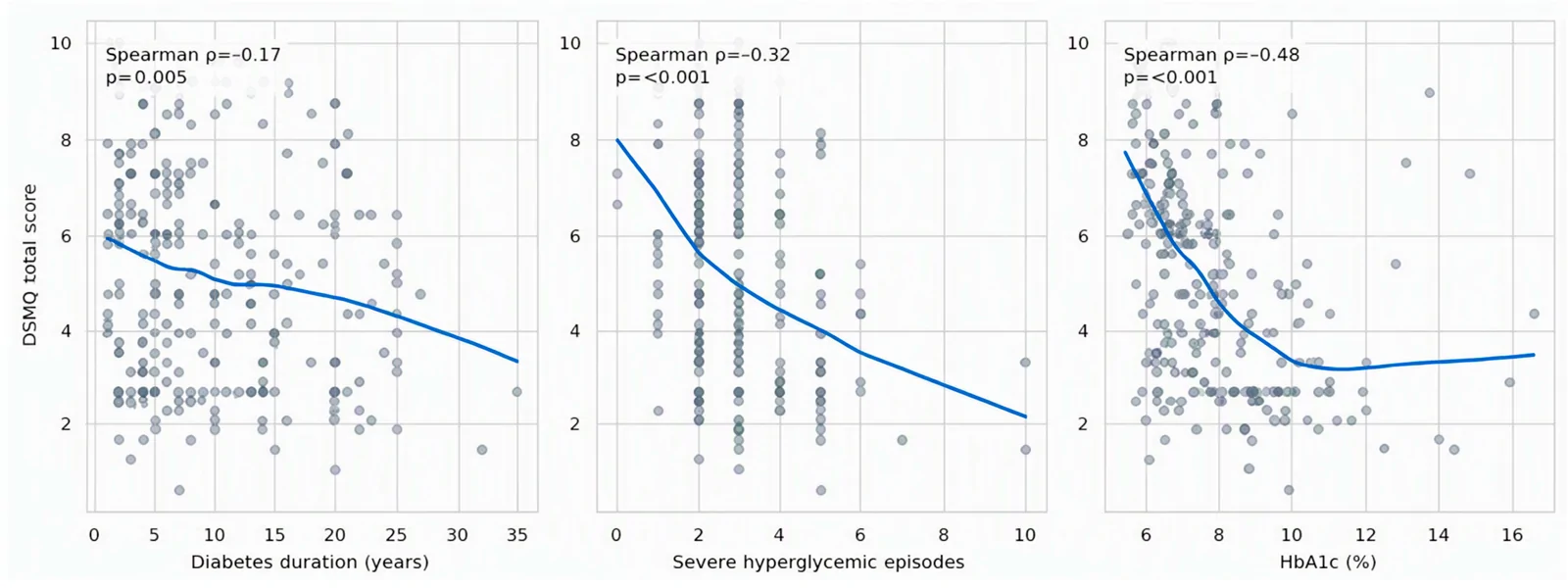
Univariable associations of the recalculated DSMQ total score with diabetes duration, the recorded severe-hyperglycemia count, and HbA1c. Curves are LOWESS smoothers with the default span (frac = 2/3), used only for descriptive visualization; they are not model-based estimates and were not used as predictors. Each dot represents one participant; dot transparency is used to improve visibility of overlapping observations. Spearman coefficients quantify monotonic association.

**Figure 3:**
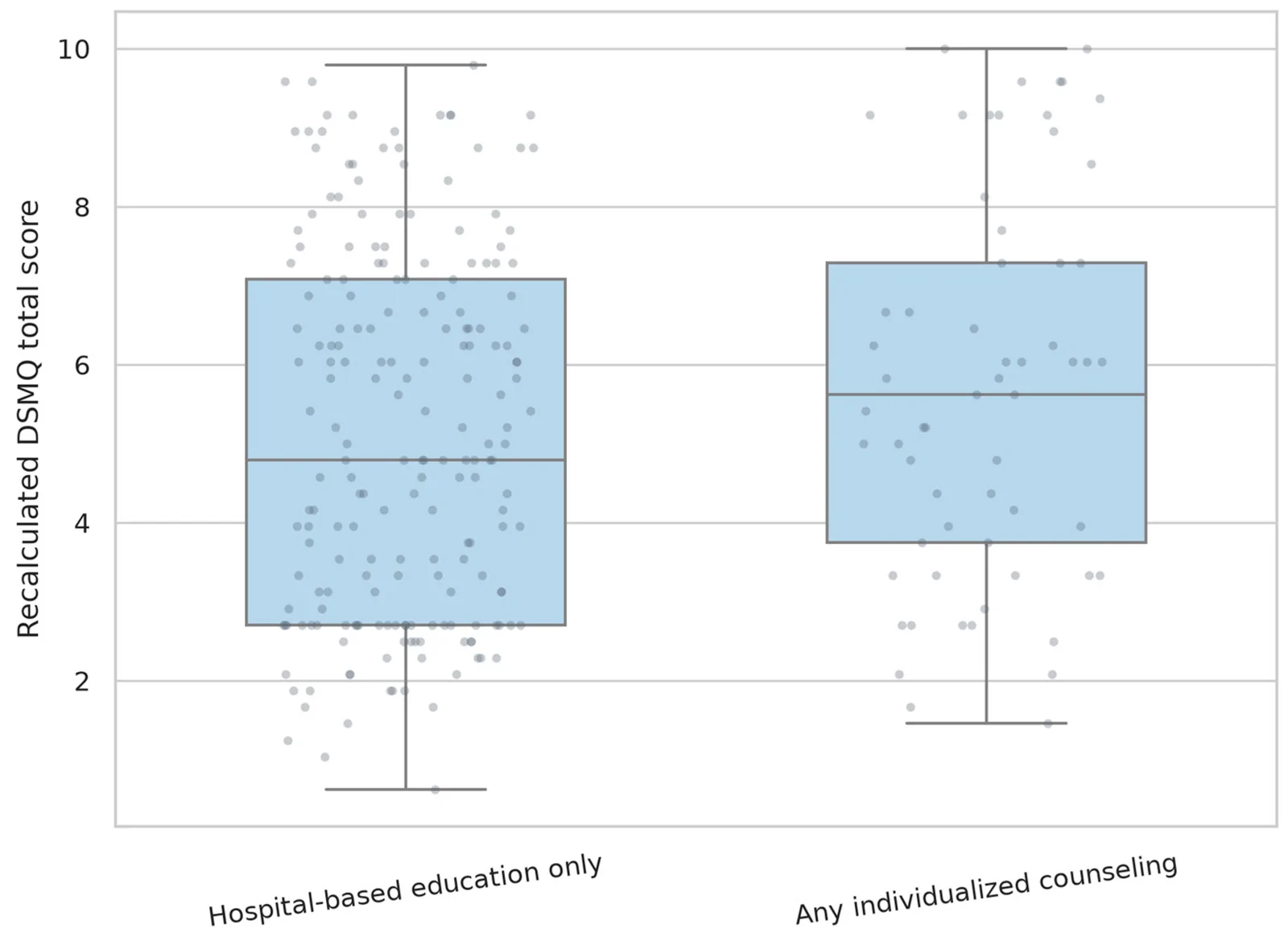
Recalculated DSMQ total score according to recorded diabetes-education modality. The single participant who received both modalities was coded as individualized counseling in the regression indicator and is shown in the individualized-counseling category for this descriptive display. Because all participants had at least one recorded modality, the figure does not estimate counseling versus no education. Gray dots represent individual participant scores; boxes indicate the interquartile range, horizontal lines indicate the median, and whiskers indicate the minimum and maximum values.

For binary variables, rank-biserial coefficients were calculated with the 0-coded group entered first; therefore, the sign depends on coding. Education modality was coded 1 for individualized counseling, including the single participant who received both modalities, and 0 for hospital-based education only. Univariable *p*-values are exploratory and were not adjusted for multiplicity. DSMQ, Diabetes Self-Management Questionnaire (Figure 2 and Figure 3).

### 3.3. Multivariable Analysis

The adjusted model included 261 participants; one participant was excluded because the sex code could not be interpreted. All VIFs were below 1.4, indicating no important multicollinearity. The Breusch–Pagan test did not indicate substantial heteroskedasticity (*p* = 0.278), and the Ramsey RESET test did not provide evidence of important quadratic misspecification (*p* = 0.097). The maximum Cook’s distance was 0.027, with no influential observation identified by conventional screening. The bounded DSMQ outcome showed no floor effect and only two observations at the upper boundary.

Only the recorded severe-hyperglycemia count remained independently associated with the continuous DSMQ total score. Each additional recorded glucose measurement > 250 mg/dL was associated with a 0.412-point lower DSMQ score (95% CI −0.621 to −0.203; *p* < 0.001). Diabetes duration was not independently associated with the corrected score (−0.032 points per year; 95% CI −0.074 to 0.009; *p* = 0.129). Individualized counseling versus hospital-based education was associated with a nonsignificant 0.463-point higher score (95% CI −0.199 to 1.124; *p* = 0.170). The coefficient is an adjusted association between modalities, not an estimate of counseling effectiveness.

The model R^2^ was 0.172, adjusted R^2^ was 0.136, and apparent RMSE was 2.14 points. After bootstrap correction, R^2^ was 0.099 and RMSE was 2.24 points. In the beta-regression sensitivity analysis, the coefficient for the recorded severe-hyperglycemia count was −0.152 on the logit mean scale (95% CI −0.232 to −0.072; *p* < 0.001), with the same direction as the linear model. These results indicate modest internal explanatory stability; they do not represent temporal or individual-level clinical prediction.

**Figure 4:**
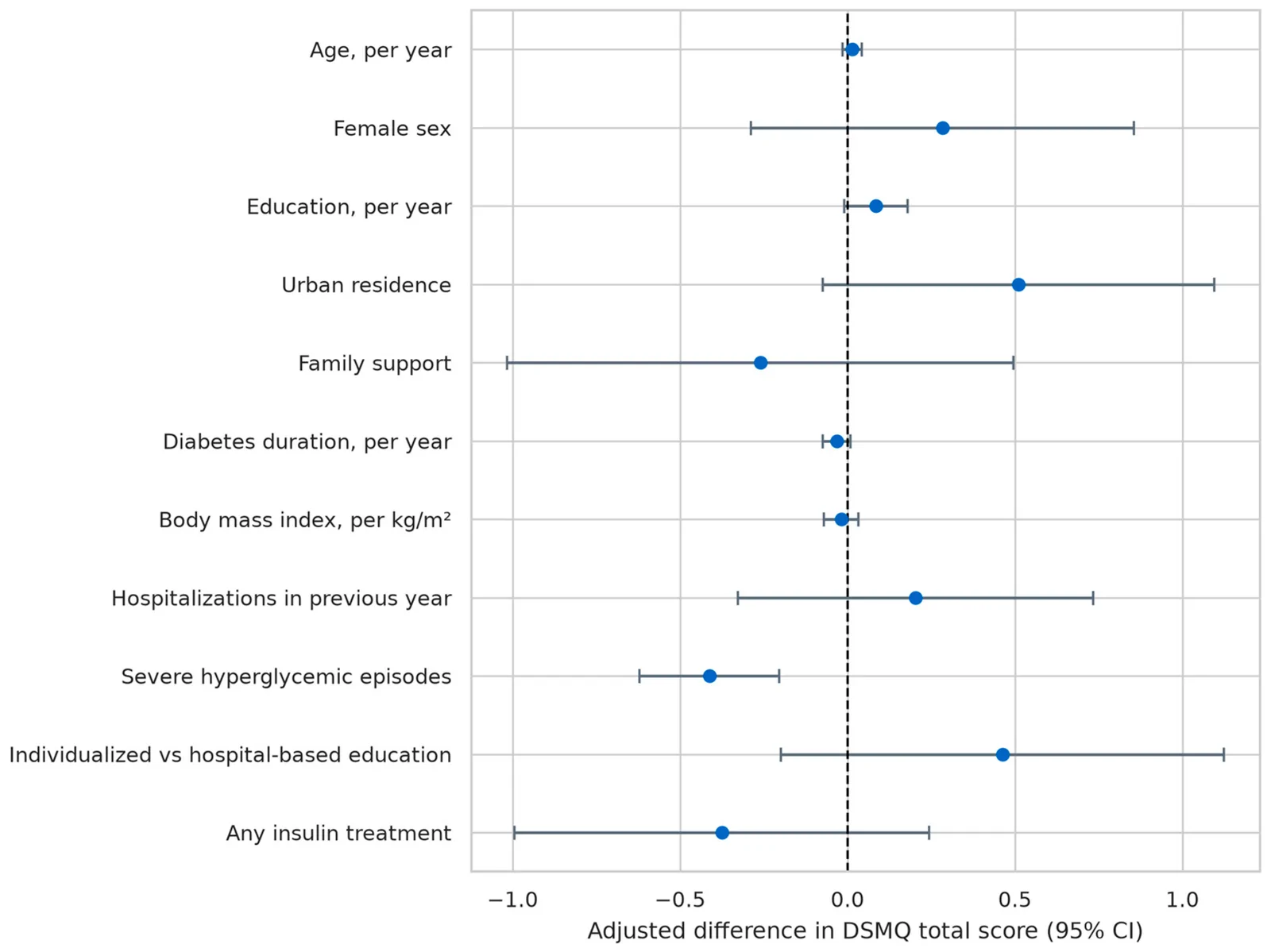
Adjusted coefficients and 95% confidence intervals from the primary multivariable linear regression model. The dashed line indicates no adjusted association.

Coefficients represent adjusted differences in DSMQ points. Binary-variable reference groups were male sex, rural residence, no recorded family support, hospital-based education only, and no insulin treatment. The education-modality indicator was 1 for individualized counseling, including the one dual-modality participant. HC3 robust standard errors were used. CI, confidence interval (Figure 4).

Visual inspection of the partial-residual plot for the count of recorded glucose measurements > 250 mg/dL showed no material departure from linearity over the observed range; therefore, a nonlinear term was not retained.

### 3.4. Secondary Median-Based Methodological Comparison

A secondary methodological comparison divided the corrected DSMQ score at the sample median (5.10; 131 participants in each group descriptively; the complete-case logistic model included 130 lower-score and 131 higher-score participants because of the missing sex code). Recorded glucose measurements > 250 mg/dL remained associated with lower odds of membership in the higher-score group (OR = 0.658; 95% CI 0.519–0.834; *p* < 0.001). Years of education also reached statistical significance in this dichotomized model (OR = 1.133 per year; 95% CI 1.036–1.239; *p* = 0.006), whereas it did not reach conventional significance in the primary continuous model (Table 4). The full adjusted model is provided in Supplementary Table S1. This instability illustrates the analytical cost of dichotomization and supports retaining the continuous score as the primary outcome [13].

## 4. Discussion

### 4.1. Principal Findings

After independent item-level DSMQ rescoring and continuous-outcome analysis, the recorded severe-hyperglycemia count was the only clinical variable that remained independently associated with the DSMQ score. Diabetes duration and the modality comparison between individualized counseling and hospital-based education were not independently associated after adjustment. The result should be interpreted as a concurrent association within the same observational dataset, not as evidence that hyperglycemia causes poorer self-management or that poorer self-management causes hyperglycemia.

The adjusted coefficient was approximately −0.41 DSMQ points per additional recorded episode. Although the estimate was statistically precise, the dataset provides no established minimal clinically important difference for the DSMQ, so the clinical importance of a one-episode difference cannot be inferred from the *p*-value alone. Because the episode variable was a preaggregated count without timestamps, observation time, or measurement-frequency information, the association may also reflect monitoring intensity, disease severity, treatment complexity, or bidirectional feedback between glycemic burden and self-management.

The inverse correlation observed between the DSMQ total score and HbA1c in our study (ρ = −0.484) was slightly stronger than the correlation reported for the DSMQ Sum Scale in the original validation study (ρ = −0.40) [3]. The direction of association was consistent, although direct comparison of magnitude requires caution because the original cohort included both type 1 and type 2 diabetes and used a more standardized temporal relationship between DSMQ assessment and HbA1c measurement.

The corrected DSMQ score remained strongly associated with concurrent HbA1c and fasting glucose, consistent with the instrument’s intended relationship with glycemic control [3,4]. These variables were not included as determinants in the main model because they were measured in the same clinical data source and may be downstream correlates of self-management. Their exclusion was conceptual rather than evidence that they are unrelated to DSMQ scores.

### 4.2. Sociodemographic Factors and Education Modality

Years of education and urban residence showed modest unadjusted associations with self-management, but neither reached conventional statistical significance after simultaneous adjustment. The education variable represented years of formal schooling and should not be interpreted as a direct measure of health literacy or diabetes knowledge. Sociodemographic factors may operate through health literacy, self-efficacy, access, and psychosocial resources rather than functioning as direct determinants [6,7,16,17,18,19,20].

Individualized counseling was not independently associated with the corrected continuous score. Because there was no no-education group, the coefficient compares two documented modalities; it cannot estimate whether education was received or whether one modality was effective. Patients may have been directed to individualized counseling because of disease severity, regimen complexity, previous self-management difficulties, or health-care utilization. The available covariates could only partially address this confounding by indication, and no causal claim regarding counseling effectiveness is justified [10,11,21,22,23,24]. The education-modality coefficient was adjusted for age in the multivariable model; however, no age-stratified analysis or age-by-modality interaction was planned, and the available data do not support conclusions regarding whether the association differs according to age.

### 4.3. Methodological Implications

The reanalysis demonstrates the importance of reconstructing questionnaire scores directly from item-level responses. The stored derived DSMQ variables were not treated as authoritative because they did not consistently reproduce the published item-level algorithm. Recalculating the total and subscale scores changed the distribution, group assignments, and inferential results. This is a methodological reanalysis of the same clinical database, not a new prospective cohort.

Retaining the DSMQ as a continuous measure avoided information loss from a sample-specific median split. Median dichotomization can reduce statistical power, create artificial differences between similar participants on opposite sides of the cut-point, and generate unstable associations [13,25,26,27]. The secondary logistic comparison is therefore presented as a methodological illustration, not as confirmation of a specific clinical threshold.

The optimism-corrected R^2^ of 0.099 and RMSE of 2.24 points indicate that the available variables explain only a modest proportion of individual variation. The beta-regression sensitivity analysis supported the direction of the hyperglycemia association, but neither model provides temporal prediction. The analysis should therefore be presented as an explanatory association study for clinicians and researchers interpreting questionnaire-based self-management data.

### 4.4. Strengths and Limitations

Strengths include complete item-level DSMQ data for 262 participants, explicit reconstruction of all scale scores, use of a continuous primary outcome, prespecified adjustment, robust standard errors, assessment of linearity and influential observations, a bounded-outcome sensitivity analysis, multicollinearity assessment, and bootstrap internal validation. The corrected total and subscale scores also showed acceptable-to-high internal consistency, with appropriate caution for short subscales.

Limitations are substantial. The cross-sectional design precludes causal inference and permits reverse causation. The documented recruitment window was available, but exact dates of DSMQ administration, laboratory sampling, consultation, and individual glucose readings were not included in the export. The severe-hyperglycemia variable was a preaggregated count without observation-time or monitoring-frequency denominators; consecutive measurements could not be deduplicated. The single-center, consecutively recruited sample limits external generalizability, and self-reported behaviors may be influenced by recall and social-desirability bias. Important potential correlates—including diabetes distress, depression, cognition, health literacy, income, medication costs, and regimen complexity—were unavailable. The education variables did not include a no-education comparison group and may reflect indication bias. One sex code was uninterpretable, and two implausible laboratory values could not be source-verified. Finally, the modest internal explanatory performance does not support individual clinical prediction.

## 5. Conclusions

In this cross-sectional cohort of adults with type 2 diabetes, the database-reported count of glucose measurements > 250 mg/dL was the most robust concurrent correlate of lower DSMQ score after item-level reconstruction. Diabetes duration and individualized versus hospital-based education were not independently associated with the corrected continuous score. The findings may help clinicians and researchers recognize when self-management assessment deserves closer review, but they do not establish cause and effect, validate a clinical threshold, or constitute a prediction model. Prospective multicenter studies should record DSMQ timing, measurement frequency, episode timestamps, psychosocial factors, health literacy, and treatment complexity so that temporal and causal hypotheses can be tested.

## Figures and Tables

**Table 1:** Baseline characteristics of the study population.

Characteristic	Value	Available *n*
Age, years	64.23 ± 10.97	262
Female sex, *n* (%)	144 (55.2)	261
Urban residence, *n* (%)	115 (43.9)	262
Married, *n* (%)	212 (80.9)	262
Family support, *n* (%)	215 (82.1)	262
0–4 years of education, *n* (%)	38 (14.5)	262
8 years of education, *n* (%)	73 (27.9)	262
12 years of education, *n* (%)	128 (48.9)	262
University education, *n* (%)	23 (8.8)	262
Diabetes duration, years	7.0 (4.0–14.0)	262
Hospitalizations in previous year	1.0 (1.0–2.0)	262
Recorded severe-hyperglycemia count	3.0 (2.0–3.0)	262
Count of recorded glucose measurements < 54 mg/dL	0.0 (0.0–0.0)	262
Body mass index, kg/m^2^	31.70 ± 5.90	262
Waist circumference, cm	108.71 ± 14.23	262
Regular physical activity, *n* (%)	185 (70.6)	262
Self-monitoring of blood glucose, *n* (%)	121 (46.2)	262
Hospital-based education only, *n* (%)	201 (76.7)	262
Any individualized counseling, *n* (%)	61 (23.3)	262
Any insulin treatment, *n* (%)	114 (43.5)	262
HbA1c, %	7.30 (6.50–8.70)	261
Fasting glucose, mg/dL	140.0 (121.0–168.0)	261
Recalculated total DSMQ score	5.26 ± 2.35	262
Recalculated total DSMQ score, median (IQR)	5.10 (3.12–7.08)	262

Data are mean ± standard deviation, median (interquartile range), or *n* (%). The available denominator is shown in Table 1. Education years were coded as 0, 4, 8, 12, or 16 years; the 12-year category includes the recorded “LICEU” entry. Female-sex percentages use *n* = 261 because one sex code was uninterpretable. HbA1c and fasting glucose use *n* = 261 after one implausible value in each variable was treated as missing. DSMQ, Diabetes Self-Management Questionnaire.

**Table 2:** Univariable associations with the recalculated DSMQ total score.

Variable	Available *n*	Measure	Estimate	*p* Value
Age	262	Spearman rho	0.015	0.815
Education years (0/4/8/12/16)	262	Spearman rho	0.150	0.015
Diabetes duration	262	Spearman rho	−0.173	0.005
Hospitalizations	262	Spearman rho	−0.061	0.324
Recorded severe-hyperglycemia count	262	Spearman rho	−0.322	<0.001
Recorded severe-hypoglycemia count	262	Spearman rho	−0.070	0.262
BMI (recalculated)	262	Spearman rho	−0.062	0.317
Waist circumference	262	Spearman rho	−0.105	0.089
HbA1c	261	Spearman rho	−0.484	<0.001
Fasting glucose	261	Spearman rho	−0.371	<0.001
Female sex (1 vs. 0)	261	Rank-biserial r	−0.097	0.179
Urban residence (1 vs. 0)	262	Rank-biserial r	−0.211	0.003
Family support (1 vs. 0)	262	Rank-biserial r	−0.021	0.818
Education modality: individualized counseling (1 vs. hospital-only)	262	Rank-biserial r	−0.136	0.107
Any insulin treatment (1 vs. 0)	262	Rank-biserial r	0.236	0.001

**Table 3:** Multivariable linear regression for the recalculated DSMQ total score.

Variable	Adjusted Coefficient	95% CI	*p* Value
Age, per year	0.014	−0.015 to 0.042	0.342
Female sex	0.283	−0.289 to 0.855	0.332
Education, per year	0.084	−0.010 to 0.179	0.081
Urban residence	0.511	−0.074 to 1.095	0.087
Family support	−0.261	−1.017 to 0.495	0.499
Diabetes duration, per year	−0.032	−0.074 to 0.009	0.129
Body mass index, per kg/m^2^	−0.019	−0.070 to 0.033	0.474
Hospitalizations in previous year	0.203	−0.327 to 0.733	0.454
Recorded severe-hyperglycemia count	−0.412	−0.621 to −0.203	<0.001
Education modality: individualized counseling (vs. hospital-only)	0.463	−0.199 to 1.124	0.170
Any insulin treatment	−0.375	−0.994 to 0.244	0.235

**Table 4:** Descriptive comparisons above and below the corrected DSMQ median.

Variable	Lower DSMQ	Higher DSMQ	*p* Value	Effect Size
Age, years	64.00 (58.50–72.00)	64.00 (57.00–74.00)	0.861	rank-biserial r = −0.013
Education, years	12.00 (8.00–12.00)	12.00 (8.00–12.00)	0.003	rank-biserial r = −0.199
Diabetes duration, years	9.00 (5.00–15.00)	7.00 (3.00–13.00)	0.024	rank-biserial r = 0.161
Hospitalizations in previous year	1.00 (1.00–2.00)	1.00 (1.00–2.00)	0.316	rank-biserial r = 0.060
Recorded severe-hyperglycemia count	3.00 (2.00–4.00)	2.00 (2.00–3.00)	<0.001	rank-biserial r = 0.328
Recorded severe-hypoglycemia count	0.00 (0.00–0.00)	0.00 (0.00–0.00)	0.099	rank-biserial r = 0.072
Body mass index, kg/m^2^	32.37 (27.89–35.52)	30.37 (27.74–33.99)	0.046	rank-biserial r = 0.142
Waist circumference, cm	111.00 (100.00–121.00)	107.00 (100.00–114.00)	0.067	rank-biserial r = 0.131
HbA1c, %	8.10 (7.00–9.60)	6.70 (6.10–7.68)	<0.001	rank-biserial r = 0.506
Fasting glucose, mg/dL	151.00 (130.00–183.00)	130.00 (112.25–150.00)	<0.001	rank-biserial r = 0.390
Female sex, *n* (%)	67 (51.5)	77 (58.8)	0.240	Cramér V = 0.073
Married, *n* (%)	105 (80.2)	107 (81.7)	0.753	Cramér V = 0.019
Family support, *n* (%)	108 (82.4)	107 (81.7)	0.872	Cramér V = 0.010
Urban residence, *n* (%)	46 (35.1)	69 (52.7)	0.004	Cramér V = 0.177
Regular physical activity, *n* (%)	89 (67.9)	96 (73.3)	0.342	Cramér V = 0.059
Current smoking, *n* (%)	36 (27.5)	26 (19.8)	0.146	Cramér V = 0.090
Alcohol use, *n* (%)	20 (15.3)	9 (6.9)	0.030	Cramér V = 0.134
Self-monitoring of blood glucose, *n* (%)	38 (29.0)	83 (63.4)	<0.001	Cramér V = 0.345
Individualized counseling, *n* (%)	26 (19.8)	35 (26.7)	0.188	Cramér V = 0.081
Any insulin treatment, *n* (%)	69 (52.7)	45 (34.4)	0.003	Cramér V = 0.185

Values are median (interquartile range) or *n* (%). The table is descriptive and methodological; the median split is not a validated clinical cut-off. For laboratory variables, one implausible value was treated as missing. DSMQ, Diabetes Self-Management Questionnaire.

## Data Availability

Aggregate results are reported in the article and its Supplementary Table. The raw database contains direct identifiers and cannot be made publicly available in its current form. A properly de-identified dataset and analysis code may be made available by the corresponding author upon reasonable request and subject to ethical and institutional approval.

## Supplementary Materials

The following supporting information can be downloaded at https://www.mdpi.com/article/10.3390/diseases14090348/s1. Table S1: DSMQ item-scoring key used for the reanalysis.

## Abbreviations

The following abbreviations are used in this manuscript:

BMI	Body mass index
CI	Confidence interval
DSMQ	Diabetes Self-Management Questionnaire
HbA1c	Glycated hemoglobin
HC3	Heteroskedasticity-consistent covariance estimator type 3
IQR	Interquartile range
RMSE	Root-mean-square error
T2DM	Type 2 diabetes mellitus
VIF	Variance inflation factor
