# Supplementary material for: Clinical Correlates of Type 2 Diabetes Self-Management in Adults: A Cross-Sectional Study
Source: Diseases. 2026 Sep 19;14(9):348. doi: 10.3390/diseases14090348 (PMC13605858; doi:10.3390/diseases14090348)
Supplement: Supplementary file 1 [file diseases-14-00348-s001.zip › diseases-4547422-supplementary.pdf]

Supplementary Table S1. Descriptive comparisons above and below the corrected DSMQ median.

Variable	Lower DSMQ	Higher DSMQ	p value	Effect size
Age, years	64.00 (58.50–72.00)	64.00 (57.00–74.00)	0.861	rank-biserial r=-0.013
Education, years	12.00 (8.00–12.00)	12.00 (8.00–12.00)	0.003	rank-biserial r=-0.199
Diabetes duration, years	9.00 (5.00–15.00)	7.00 (3.00–13.00)	0.024	rank-biserial r=0.161
Hospitalizations in previous year	1.00 (1.00–2.00)	1.00 (1.00–2.00)	0.316	rank-biserial r=0.060
Severe hyperglycemic episodes	3.00 (2.00–4.00)	2.00 (2.00–3.00)	<0.001	rank-biserial r=0.328
Severe hypoglycemic episodes	0.00 (0.00–0.00)	0.00 (0.00–0.00)	0.099	rank-biserial r=0.072
Body mass index, kg/m²	32.37 (27.89–35.52)	30.37 (27.74–33.99)	0.074	rank-biserial r=0.128
Waist circumference, cm	111.00 (100.00–121.00)	107.00 (100.00–114.00)	0.067	rank-biserial r=0.131
HbA1c, %	8.10 (7.00–9.60)	6.70 (6.10–7.67)	<0.001	rank-biserial r=0.506
Fasting glucose, mg/dL	151.00 (130.00–183.00)	130.00 (112.25–150.00)	<0.001	rank-biserial r=0.390
Female sex, n (%)	67 (51.5)	77 (58.8)	0.240	Cramér V=0.073
Married, n (%)	105 (80.2)	107 (81.7)	0.753	Cramér V=0.019
Family support, n (%)	108 (82.4)	107 (81.7)	0.872	Cramér V=0.010
Urban residence, n (%)	46 (35.1)	69 (52.7)	0.004	Cramér V=0.177
Regular physical activity, n (%)	89 (67.9)	96 (73.3)	0.342	Cramér V=0.059
Current smoking, n (%)	36 (27.5)	26 (19.8)	0.146	Cramér V=0.090
Alcohol use, n (%)	20 (15.3)	9 (6.9)	0.030	Cramér V=0.134
Self-monitoring of blood glucose, n (%)	38 (29.0)	83 (63.4)	<0.001	Cramér V=0.345
Individualized counseling, n (%)	26 (19.8)	35 (26.7)	0.188	Cramér V=0.081
Any insulin treatment, n (%)	69 (52.7)	45 (34.4)	0.003	Cramér V=0.185

This table is descriptive and sensitivity-oriented. The median split is not a validated clinical cut-off. DSMQ, Diabetes Self-Management Questionnaire.
